# Snake bite on scrotum – a case report

**DOI:** 10.4314/pamj.v10i0.72236

**Published:** 2011-10-20

**Authors:** Anjum Arshad, Mateen Azfar, Husain Munawwar, Usmani A Jawed

**Affiliations:** 1 Department of Forensic medicine and Toxicology, Jawaharlal Nehru Medical College, Aligarh Muslim University, Aligarh, Uttar Pradesh, India

**Keywords:** Snake bite, scrotum, India

## Abstract

A 22-year old man was bitten by a snake on his scrotum. This interesting and unusual case occurred in the rural area of District Aligarh, India. The uniqueness of the case lies in the fact that scrotum is an extremely rare and unusual site for snake bite. Further, with negligible local signs of envenoming the patient presented with classical signs of neurotoxicity. Due to numerous superstitions associated with snake bite, the patient was treated with traditional home made ointment before coming to hospital. The authors realized that the case may be brought to the notice of the readers because the scrotal bite by the snake with no local signs of envenomation is the first reported case.

## Background

Since the dawn of civilization, snakes have inspired a mystic feeling of good and evil in human mind. In India popular folklores and deep rooted superstitions have put the asp in some places at the height of God and somewhere at the depth of hell as the evil incarnate. In India, snakes are found everywhere from 12,000 feet altitude of the Himalayas down to Cape Comorin, but different areas have different species preponderance. India is inhabited by more than 60 species of venomous snakes out of which only four have been popularly known to be dangerously poisonous to man; cobra, common krait, Russell viper and Saw Scaled Viper [1]. In India each year approximately 200,000 number of cases of snake bite are reported, out of which 45,000 to 50,000 succumb to death [2]. The problem is so under-rated that it was only added to WHO′s list of neglected tropical diseases in April 2009 [2]. Many cases are non poisonous in nature, but emotional calamity and fright render them disastrous to the victims and their families. The most frequent site of bites is the lower extremity [3]. However, till date snake bite on the scrotum with negligible local signs has not been reported in scientific journals.

## Case presentation

**Figure 1:**
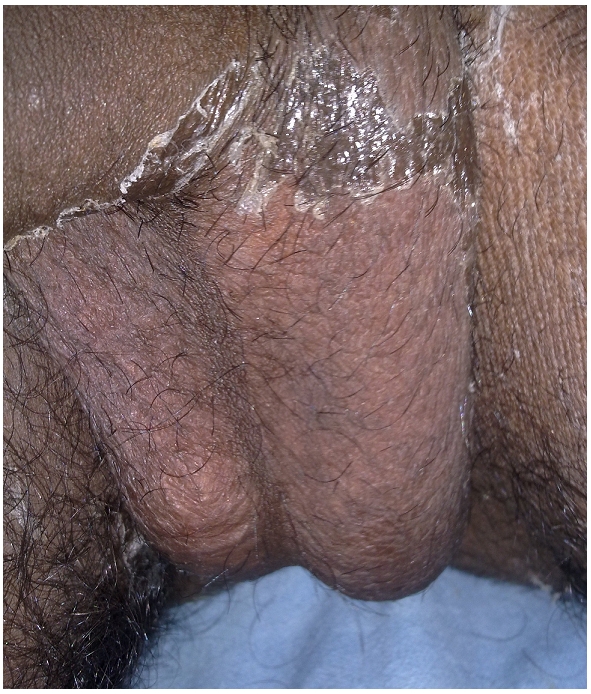
Homemade ointment rubbed over the scrotum and on bitten part (arrow) as a measure of first line of treatment in a patient victim of snake bite on scrotum

The patient Mr ABC (patient details kept anonymous), 22 years old male, is a resident of Iglas, a small town which is about 25 km from the main city Aligarh. In the city, adequate facilities are available for treatment of all sorts of snake bite, including critical care management in the worst eventuality. However, due to ritual-based customs and dogma, the victim was treated by home-made ointment (Figure 1). The patient was bitten by the snake at about 12.30 am and hence precious six hours were lost while the patient was shunted from local quack to “tantrik” who generally claim to have cure for all sorts of illnesses. Finally, the patient was brought to the Emergency Section of Jawaharlal Nehru Medical College Hospital, Aligarh, at 06:30 am.

According to the history given by the relatives of the patient, and which was substantiated and authenticated by the patient himself upon recovery, he was sleeping on the roof-top of his mud constructed house wearing a “lungi” (the loin cloth). At 12:30 am he felt something crawling up his thighs. By the time his reflexes worked the snake bit him on the scrotum and he felt immediate pain followed by little ooze of blood from the bite site. Immediately, driven by fright he caught the snake and threw it violently on the roof. It was killed by stamping over. No effort was made to identify it whether it was the poisonous or non poisonous variety. In India, cutting across all distinction between rich or poor, educated or illiterate, it is an ingrained belief that belligerent snake should be burnt after killing as the picture of its killer gets recorded in the eyes of it. Later, people believe the female snake particularly distinguish the killer by seeing the eyes of the killed snake and take revenge. Hence the snake was not available to identify its species.

### Signs and symptoms

**Figure 2:**
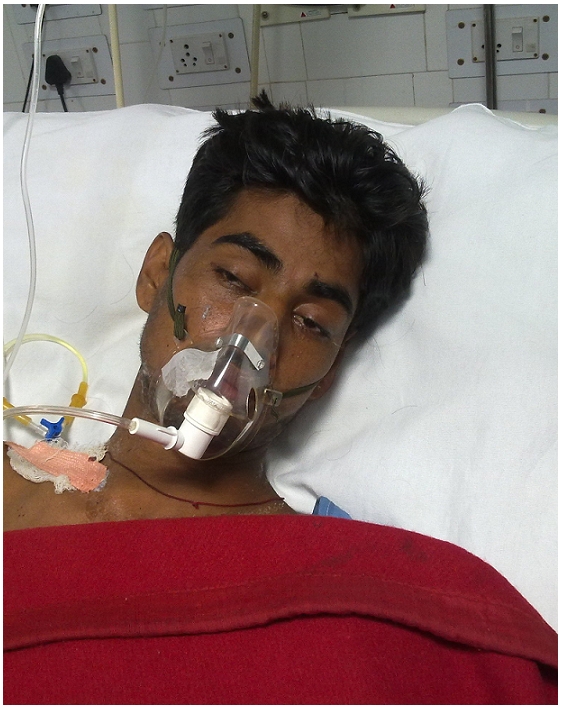
Patient victim of a snake bite on scrotum, admitted in ICU with typical neurotoxic symptoms (ptosis and inability to hold neck)

The patient presented with a classic sings of neurotoxicity. He had ptosis, drooling of saliva, sluggishness, apathy, disorientation, slurring of speech, inability to hold neck and difficulty in respiration (Figure 2). On examination of bite site, there was slight redness on inferior aspect of scrotum without any features of swelling, bruising, blistering, local bleeding, etc. The patient was shifted to ICU for support of mechanical ventilation and was promptly administered the Anti Snake Venom (ASV). The adjunct therapy included atropine, neostigmine, antibiotic, i.v. fluids etc. This standard treatment continued, and he was weaned away from the ventilator after 36 hours and later shifted to ward for observation.

The patient made a remarkable recovery and was discharged without any sequelae after ten days of hospital admission. He is asked to report after 1 week for further evaluation.

### Proposal

The patient was a married man who tied the nuptial knot three months before the unfortunate incident. His wife was not pregnant at the time of the tragic occurrence. It is justifiably proposed that the patient would be followed up periodically to assess his virility and fertility. Regular examination of sperm count and normalcy would be done with the consent of the patient. A second reporting shall be done after adequate follow-up of the case.

## Conclusions

In Indian subcontinent, people particularly in rural areas sleep on roof tops, which make them prone to Snake bites. Scrotum is a very rare and unusual site for snake bite. There are numerous superstitions associated with snakes and people still rely on traditional measures as first line of treatment, and thus valuable time is lost before patient is brought to hospital.
